# Development of Z_HPV16E7_- granzyme B immunoaffitoxin: dual mechanisms targeting hpv16-positive cervical cancer through epithelial-mesenchymal transition inhibition and cell death

**DOI:** 10.3389/fimmu.2025.1616715

**Published:** 2025-07-21

**Authors:** Xiaochun Tan, Jiani Yang, Yanheng Li, Kairong Wan, Sicong Feng, Xishang Jing, Zhenyun Xie, Lifang Zhang, Wenshu Li

**Affiliations:** ^1^ Institute of Molecular Virology and Immunology, Department of Microbiology & Immunology, School of Basic Medical Sciences, Wenzhou Medical University, Wenzhou, China; ^2^ Department of Laboratory Medicine, The First Hospital of Jiaxing, Affiliated Hospital of Jiaxing University, Jiaxing, China; ^3^ Department of Laboratory Medicine, Nanjing University of Chinese Medicine, Yancheng TCM Hospital, Yancheng, China; ^4^ Communication Department, Technical University of Valencia, Valencia, Spain

**Keywords:** HPV16E7, affibody, GrB, cervical cancer, EMT, cell death

## Abstract

**Introduction:**

Cervical cancer, predominantly caused by high-risk human papillomavirus (HPV), remains a major global health challenge. HPV16 is the most prevalent type, and its oncoprotein E7 promotes epithelial-mesenchymal transition (EMT), a critical step in metastasis. Current therapies for HPV16-related cancers are often insufficient, highlighting the need for targeted treatments. We engineered a novel immunotoxin, ZHPV16E7-GrB, by fusing an HPV16E7-specific affibody (ZHPV16E7) with the cytotoxic immune effector granzyme B (GrB). This construct was designed for precise targeting and therapeutic activity against HPV16-positive cervical cancer cells.

**Methods:**

ZHPV16E7-GrB was engineered, expressed in *E. coli*, and purified. Binding specificity was assessed via ELISA and immunofluorescence using HPV16-positive (SiHa, CaSki), HPV18-positive (HeLa), and HPV-negative (C33A) cervical cancer cells. Functional assays evaluated cell viability (LDH release, luminescence), migration (Transwell), EMT markers (Western blot for E-cadherin, N-cadherin, Vimentin, Snail), apoptosis (TUNEL, flow cytometry, caspase activation), and pyroptosis (SYTOX Green uptake, cytokine release ELISA, GSDME cleavage). Caspase-3 knockdown and in vitro cleavage assays determined pyroptosis mechanisms.

**Results:**

ZHPV16E7-GrB exhibited strong binding specificity for HPV16E7. It significantly inhibited cell growth and suppressed EMT in HPV16-positive cells, evidenced by reduced migration and invasion, downregulation of Vimentin and Snail, increased E-cadherin, and decreased N-cadherin expression. Furthermore, ZHPV16E7-GrB induced apoptosis via caspase-3/caspase-8 activation and triggered pyroptosis through direct cleavage of gasdermin E (GSDME), independent of caspase-3, accompanied by membrane rupture and proinflammatory cytokine release. Crucially, ZHPV16E7-GrB demonstrated selective toxicity, effectively killing HPV16-positive cells while sparing non-HPV16-positive cells, minimizing off-target effects.

**Discussion:**

This study highlights the dual mechanism of ZHPV16E7-GrB, inhibiting EMT and inducing cell death (apoptosis and pyroptosis). These findings demonstrate its significant promise as a targeted therapeutic agent for HPV16-associated cervical cancer, addressing critical unmet needs in current treatment strategies.

## Introduction

Cervical cancer is a leading cause of cancer-related deaths among women worldwide, with persistent infection by high-risk human papillomavirus (HPV) types being the primary cause. Among these, HPV16 is the most prevalent, responsible for approximately 50% of all cervical cancer cases (1). The oncogenes E6 and E7, expressed by HPV, play a key role in the malignant transformation of cervical cells. Specifically, E7 binds to the retinoblastoma protein (pRb), destabilizing the pRb-E2F complex and promoting uncontrolled cell cycle progression. This disruption leads to abnormal cell proliferation and genomic instability, hallmark features of HPV-induced carcinogenesis (2). Furthermore, the HPV16 E7 oncoprotein is strongly associated with epithelial-mesenchymal transition (EMT), a process that allows cancer cells to lose their epithelial characteristics and acquire mesenchymal traits, facilitating migration, invasion, and metastasis (3). Targeting E7 could effectively inhibit EMT, potentially preventing the spread of cancer. Although vaccines for HPV16 and other high-risk types are available, they primarily prevent infection rather than treating existing cervical cancer (4). Additionally, HPV-associated cervical cancer is difficult to treat due to its tendency to recur, metastasize, and develop resistance to conventional therapies like chemotherapy and radiotherapy. Therefore, novel therapeutic strategies, particularly targeted therapies against HPV16, are urgently needed to improve treatment outcomes and survival rates for women with cervical cancer (5).

Targeted therapeutics rely on precise drug delivery systems comprising a targeting moiety and a cytotoxic payload (6, 7). While monoclonal antibodies (mAbs) are widely used as targeting agents (8–11), affibodies—small engineered proteins derived from the “Z” domain of *Staphylococcus* Protein A—offer advantages such as enhanced tumor penetration and reduced immunogenicity (12, 13). Their simple phage display-based selection enables rapid development of high-affinity binders against various targets, including insulin (Z_insulin_), TNF-α (Z_TNF-α_), and HER2 (Z_HER2_) (14–16). In our previous study, we developed Z_HPV16E7_, an affibody molecule specifically targeting the HPV16 E7 oncoprotein. Z_HPV16E7_ significantly inhibited cervical cancer growth by blocking E7-pRb signaling, wherein HPV16 E7 inactivates tumor suppressor pRb, driving uncontrolled proliferation; disruption of this interaction restores pRb’s growth-suppressive function to halt tumor progression (13, 17). Moreover, while HPV16 E6/E7 oncoproteins promote EMT through cadherin dysregulation (specifically downregulating E-cadherin and upregulating N-cadherin) (18), Z_HPV16E7_’s ability to suppress EMT remains unverified. Given that E7 pathway inhibition alone may be insufficient for complete tumor eradication, we engineered Z_HPV16E7_-GrB—a drug conjugate combining Z_HPV16E7_ with cytotoxic granzyme B (GrB). Several drug conjugates have been clinical used with encouraging results, especially, a conjugation of anti-HER2 antibody and cytotoxic topoisomerase I inhibitor, was approved in China in 2023 (19). Cervical cancer is of high recurrence and high metastasis cancer type, and the new attempt of affibody-based drug conjugate for targeted therapy of cervical cancer should be an efficient strategy.

GrB is a potent serine protease with high cytotoxic activity, primarily known for inducing caspase-dependent apoptosis in target cells. It is produced by immune cells such as cytotoxic T lymphocytes (CTLs) and natural killer (NK) cells, playing a key role in immune responses against infected or cancerous cells (20). GrB enters target cells and activates the caspase cascade, leading to programmed cell death through apoptosis. However, recent studies have expanded our understanding of GrB’s functions. In addition to its apoptotic activity, GrB has been shown to cleave gasdermin E (GSDME), a member of the gasdermin protein family, to produce the active GSDME-N fragment which is associated with pyroptosis—a form of programmed cell death characterized by cell swelling, membrane rupture, and the release of proinflammatory cytokines. Pyroptosis is typically executed by pore-forming proteins like GSDMA, B, C, D, and E, and has recently emerged as a crucial mechanism in cancer immune responses. This cleavage of GSDME by GrB can induce pyroptosis, thus activating a potent antitumour immune response. Consequently, GrB can induce cytolysis through a variety of mechanisms, including apoptosis, pyroptosis, or a combination of both, providing a versatile approach to immune-mediated tumour destruction (21–23). In our previous studies, we introduced specific mutations into wild-type Granzyme B (GrB(W)) to generate an optimized Granzyme B (GrB(O)), which eliminated nonspecific binding to cells while maintaining its cytotoxic activity, and subsequently conjugated it with Z_HPV16E7_ to form the immunoaffitoxin Z_HPV16E7_-GrB. This conjugate retained the high specificity of Z_HPV16E7_ for HPV16-positive cervical cancer cells while effectively delivering the cytotoxic effects of GrB (24, 25). However, whether Z_HPV16E7_-GrB can simultaneously induce both apoptosis and pyroptosis in target cells remains to be experimentally verified.

This study aims to investigate the dual mechanisms of the Z_HPV16E7_-GrB conjugate: First, whether the Z_HPV16E7_ moiety suppresses EMT by targeting HPV16 E7; and second, whether the GrB payload induces cytotoxicity through both apoptosis and pyroptosis – specifically, by examining pyroptosis via direct cleavage of GSDME. By addressing these questions, this study will delineate the compound’s bifunctional activity and advance its therapeutic potential.

## Materials and methods

### Materials

The following reagents were used: restriction enzymes (*BamHI*, *HindIII*, *SmaI*), DNA marker, and protein markers (MBI Ferments, Burlington, Ontario, CA); EndoFree Plasmid Maxi Kits, IPTG, and Ni-NTA agarose (QIAGEN, Hilden, Germany); RPMI-1640 medium, FBS, penicillin, and streptomycin (Gibco, USA); Lipofectamine™ 3000 reagent (Thermo, USA); DeadEnd™ Fluorometric TUNEL System (Promega, USA); Annexin V-FITC/PI apoptosis kit (MultiSciences, China); LDH Release Assay Kit, CellTiter-Lumi™ Luminescent Cell Viability Assay Kit (Beyotime, China); human IL-18, IL-1β, and HMGB-1 ELISA kits (Elabscience, China); and anti-FLAG Affinity gel purified kit (Sangon Biotech, China). Primary antibodies used included: anti-His tag (66005-1-Ig, Proteintech); anti-GSDME, anti-GrB, anti-Bax, anti-Caspase-3, and anti-FLAG (Abcam, ab215191, ab208586, ab32503, ab32351, ab205606); anti-Caspase-8, and anti-Snail (Cell Signaling Technology, #9496, #3879); anti-GAPDH (Good Here, AB-P-R 001); anti-HPV16E7, anti-E-Cadherin, N-cadherin, anti-Vimentin, and anti-Bcl2 (Santa Cruz, sc-6981, sc-8426, sc-53488 sc-6260, sc-56018). Secondary antibodies included HRP-labelled goat anti-rabbit IgG, HRP-labelled goat anti-mouse IgG, Cy3-labelled goat anti-mouse IgG, and FITC-labelled goat anti-rabbit IgG (Beyotime, A0208, A0216, A0521, A0562). The HPV16E7 protein without the His tag was prepared in our laboratory.

### Plasmids and cell culture

Plasmids containing coding sequences, including *p*ET21a (+)/Z_HPV16E7_-GrB, *p*ET21a (+)/Z_HPV16E7_, and *p*ET21a (+)/Z_WT_-GrB (wild-type Z_WT_ affibody without affinity screening, used as an untargeted control), were constructed and maintained in our laboratory. The plasmid *p*CMV-3×FLAG-GSDME (human)-Neo (P68941) was sourced from MiaoLingPlasmid, China. The following human cervical cancer cell lines were used: SiHa (HPV16-positive, ATCC HTB-35), CaSki (HPV16-positive, ATCC CRM-CRL-1550), HeLa (HPV18-positive, ATCC CCL-2.1, as the HPV type control), and C33A (HPV-negative, ATCC HTB-31, as the HPV-free control). These cell lines were purchased from the Cell Bank of the Shanghai Institutes for Biological Sciences, Chinese Academy of Sciences, and cultured in RPMI-1640 medium supplemented with 100 mg/L penicillin and streptomycin and 10% fetal bovine serum (FBS) at 37°C in a 5% CO_2_ humidified atmosphere.

### GrB optimization and biological activity assay

We first systematically optimized its amino acid sequence (GenBank No. AAA75490.1) through two critical modifications: (i) mutation of key residues to prevent protease inhibition, and (ii) replacement of polar amino acids with nonpolar counterparts to reduce nonspecific adsorption. The engineered GrB gene was subsequently synthesized and directionally cloned into the *p*cDNA3.1(+) vector using *HindIII* and *BamH I* restriction sites. For functional analysis, we performed transient transfection of SiHa cells in 6-well plates using 1 μg of the recombinant *p*cDNA3.1/GrB plasmid per well with Lipofectamine™ 3000, with protein expression confirmed after 24-hour incubation by both Western blot and indirect immunofluorescence. Finally, to fully characterize GrB’s biological effects, we implemented a multi-modal analytical approach: (1) quantitative assessment of apoptosis induction using flow cytometry combined with TUNEL assay, (2) detection of GSDME cleavage (a key executioner of pyroptosis) by Western blot, followed by precise quantification of protein band intensities using ImageJ software to ensure rigorous data analysis.

### Preparation and identification of Z_HPV16E7_-GrB

The immunoaffitoxin Z_HPV16E7_-GrB was engineered in our laboratory (25) as a fusion protein comprising three key components: (1) an HPV16E7-specific affibody (Z_HPV16E7_), (2) human granzyme B (GrB), and (3) a flexible peptide linker (Gly_4_Ser)_3_ connecting these domains, with an N-terminal 6× His tag to facilitate purification. For recombinant protein expression, we transformed *E. coli* BL21(DE3) competent cells with three plasmid constructs: *p*ET21a (+)/Z_HPV16E7_-GrB, *p*ET21a (+)/Z_HPV16E7_, and *p*ET21a (+)/Z_WT_-GrB). Each transformation used 100ng plasmid DNA per 50 μL aliquot of competent cells. Following transformation, positive clones were selected and expanded in LB medium. Protein expression was then induced with 1 mM IPTG for 8 hours at 37°C, and the target proteins were purified via Ni²^+^-chelated affinity chromatography. The purified proteins were verified by both SDS-PAGE and Western blot analysis. Finally, the three-dimensional structure of the immunoaffitoxin was predicted using the GalaxyWEB server (http://galaxy.seoklab.org/).

### Evaluation of binding affinity

The binding affinity and targeting specificity of Z_HPV16E7_-GrB were evaluated using two approaches: an ELISA assay to assess its affinity for the HPV16E7 protein and an indirect immunofluorescence assay to determine its targeting ability toward HPV16-positive cells. For the ELISA assay, high-adhesion 96-well flat-bottom microtiter plates were first coated overnight at 4°C with 100 μL of HPV16E7 protein (10 μg/mL, His-tag-free), followed by blocking with 5% skim milk in PBST at 37°C for 2 hours. The wells were then incubated overnight at 4°C with 300 μg/mL of Z_HPV16E7_-GrB, Z_HPV16E7_, or Z_WT_-GrB. Afterward, an anti-His tag monoclonal antibody (diluted 1:5000) was applied at 37°C for 2 hours, followed by a 1.5-hour incubation with HRP-conjugated anti-mouse IgG (diluted 1:5000 in PBST) to detect bound antibodies. Finally, the chromogenic substrate TMB was added, and the reaction was allowed to develop for 15 minutes at 37°C. The color development was measured at 450 nm using an automated ELISA plate reader (Bio-Tek ELx800). To further evaluate the targeting ability of Z_HPV16E7_-GrB *in vitro*, an indirect immunofluorescence assay was performed. SiHa, CaSki, HeLa, and C33A cells were seeded on glass slides and co-cultured with 100 μg/mL of Z_HPV16E7_-GrB, Z_HPV16E7_, or Z_WT_-GrB for 24 hours. After incubation, the cells were fixed with 4% paraformaldehyde at room temperature for 10 minutes, followed by overnight blocking at 4°C in PBS containing 5% FBS. Subsequently, the slides were incubated overnight at 4°C with primary antibodies—either anti-GrB or anti-HPV16E7 (both diluted 1:1000). The following day, the samples were incubated for 1 hour at 37°C with secondary antibodies: FITC-conjugated goat anti-rabbit IgG (H+L) or Cy3-conjugated goat anti-mouse IgG (H+L) (both diluted 1:1000). Nuclei were counterstained with DAPI at 37°C for 5 minutes. Finally, fluorescence signals were analyzed using a Nikon fluorescence microscope (Japan).

### Cell viability assay

To evaluate cell viability, 8,000 cells per well were seeded in 96-well plates. After a 24-hour adhesion period, cells were treated for 72 hours with either 100 μg/mL of Z_HPV16E7_-GrB, Z_HPV16E7_, or Z_WT_-GrB, or 50 μg/mL oxaliplatin (positive control). Cell viability was then assessed using both the LDH Release Assay Kit and CellTiter-Lumi™ Steady-Glo Detection Kit according to the manufacturers’ protocols.

### Analysis of EMT

To investigate whether Z_HPV16E7_-GrB could reverse EMT in cervical cancer cells, we performed a comprehensive analysis combining Transwell migration assays with EMT marker detection. First, we assessed cellular migration using Matrigel-coated Transwell inserts (8.0 µm pore size; Corning) in 24-well plates. After pretreating cells for 24 hours with 100 μg/mL of either Z_HPV16E7_-GrB, Z_HPV16E7_, or Z_WT_-GrB, we seeded 1×10^5^ cells in serum-free medium into the upper chamber, while adding 600 μL of complete medium with 10% FBS to the lower chamber as a chemoattractant. Following 24-hour incubation, we carefully removed non-migrated cells from the upper membrane surface using PBS-moistened cotton swabs. The migrated cells on the lower surface were then fixed with 4% paraformaldehyde, stained with 0.4% crystal violet, and quantified by counting cells in five random 20× microscopic fields per insert. To directly examine EMT-related molecular changes, we performed Western blot analysis on cells treated identically for 24 hours. We probed for key EMT markers including epithelial markers (E-cadherin and N-cadherin), mesenchymal marker Vimentin, and transcription factor Snail.

### Apoptosis analysis

To comprehensively assess Z_HPV16E7_-GrB-induced apoptosis, we employed a multi-method approach combining TUNEL assay, flow cytometry, and Western blot analysis. Cervical cancer cells were treated with 100 μg/mL of Z_HPV16E7_-GrB, Z_HPV16E7_, or Z_WT_-GrB for 18 hours before analysis. First, apoptosis was detected via the DeadEnd™ Fluorometric TUNEL System, with TUNEL-positive cells quantified under fluorescence microscopy. Second, treated cells were collected and resuspended at 3 × 10^6^ cells/mL for flow cytometry using Annexin V-FITC and propidium iodide staining on a FACSCanto II system (BD, USA), followed by data analysis with FlowJo V10.1. Third, Western blot analysis was performed to evaluate key apoptotic regulators, including pro-apoptotic Bax, anti-apoptotic Bcl-2, and the activated forms of caspase-8 and caspase-3, providing mechanistic insights into the apoptotic pathway triggered by Z_HPV16E7_-GrB.

### Pyroptosis analysis

To systematically investigate Z_HPV16E7_-GrB-induced pyroptosis, we employed an integrated analytical approach with the following experimental design: target cells were treated under standard culture conditions with 100 μg/mL of Z_HPV16E7_-GrB, Z_HPV16E7_, or Z_WT_-GrB for 24 hours, followed by multiparametric evaluation. Initial assessment was performed using 1 μM SYTOX Green nucleic acid stain (37°C for 10 minutes) combined with fluorescence microscopy to quantify plasma membrane integrity loss as an indicator of early pyroptotic membrane rupture, while simultaneously measuring released inflammatory mediators (IL-1β, IL-18, and HMGB1) in supernatants using ELISA kits according to the manufacturer’s protocols. Subsequent microscopy analysis documented characteristic pyroptotic morphology including cellular swelling, membrane blebbing, and lysis, with Western blot analysis confirming GSDME cleavage as molecular evidence of pyroptosis. This comprehensive assessment strategy examined: (1) membrane integrity (SYTOX Green), (2) cytokine release (ELISA), (3) cellular morphology, and (4) molecular markers (GSDME cleavage).

### Characterization of pyroptosis under caspase-3 knockdown conditions

To examine whether Z_HPV16E7_-GrB-induced pyroptosis requires caspase-3 activity, we performed siRNA-mediated knockdown of caspase-3 in SiHa cells. Briefly, cells in 6-well plates were transfected with 1μg of caspase-3-specific siRNA (5’-CCGACAAGCUUGAAUUUAUTT-3’) or control nontargeting siRNA (5’-UUCUCCGAACGUGUCACGUTT-3’) using Lipofectamine™ 3000. 48 hours post-transfection, Western blot analysis was performed to verify the knockdown efficiency of caspase-3. These caspase-3-knockdown cells were then exposed to Z_HPV16E7_-GrB (100 μg/mL) for 24 hours, after which pyroptosis was evaluated through multiple parameters: GSDME cleavage by Western blot analysis, membrane integrity by SYTOX Green uptake, inflammatory cytokine release via ELISA, - all performed according to our established pyroptosis assessment protocol.

### GSDME cleavage assay

To investigate whether Z_HPV16E7_-GrB can directly cleave GSDME, we first established a eukaryotic expression system for FLAG-tagged GSDME. This was achieved by transfecting SiHa cells with the GSDME plasmid using Lipofectamine™ 3000 for 48 hours, followed by Western blot confirmation of successful expression. Following this, we purified the FLAG-GSDME protein from cell lysates using an anti-FLAG Affinity Gel kit, and verified the protein by both SDS-PAGE and Western blot analysis. Subsequently, to examine direct cleavage activity, we incubated 100 μg of the purified FLAG-GSDME with 100 μg of Z_HPV16E7_-GrB, Z_HPV16E7_, or Z_WT_-GrB for 1 hour in reaction buffer (50 mM Tris-HCl, pH 8.0, 150 mM NaCl), Western blot analysis was then performed to detect GSDME cleavage fragments.

### Statistical analysis

Data are presented as means ± standard deviations (SD), unless otherwise specified. Statistical comparisons were exclusively performed within the same cell line. These comparisons were conducted using one-way ANOVA, followed by the least significant difference (LSD) test. A P value of < 0.05 was considered statistically significant. All statistical analyses were performed using SPSS 22.0 software.

## Results

### Biological activity of the optimized GrB

**Figure 1:**
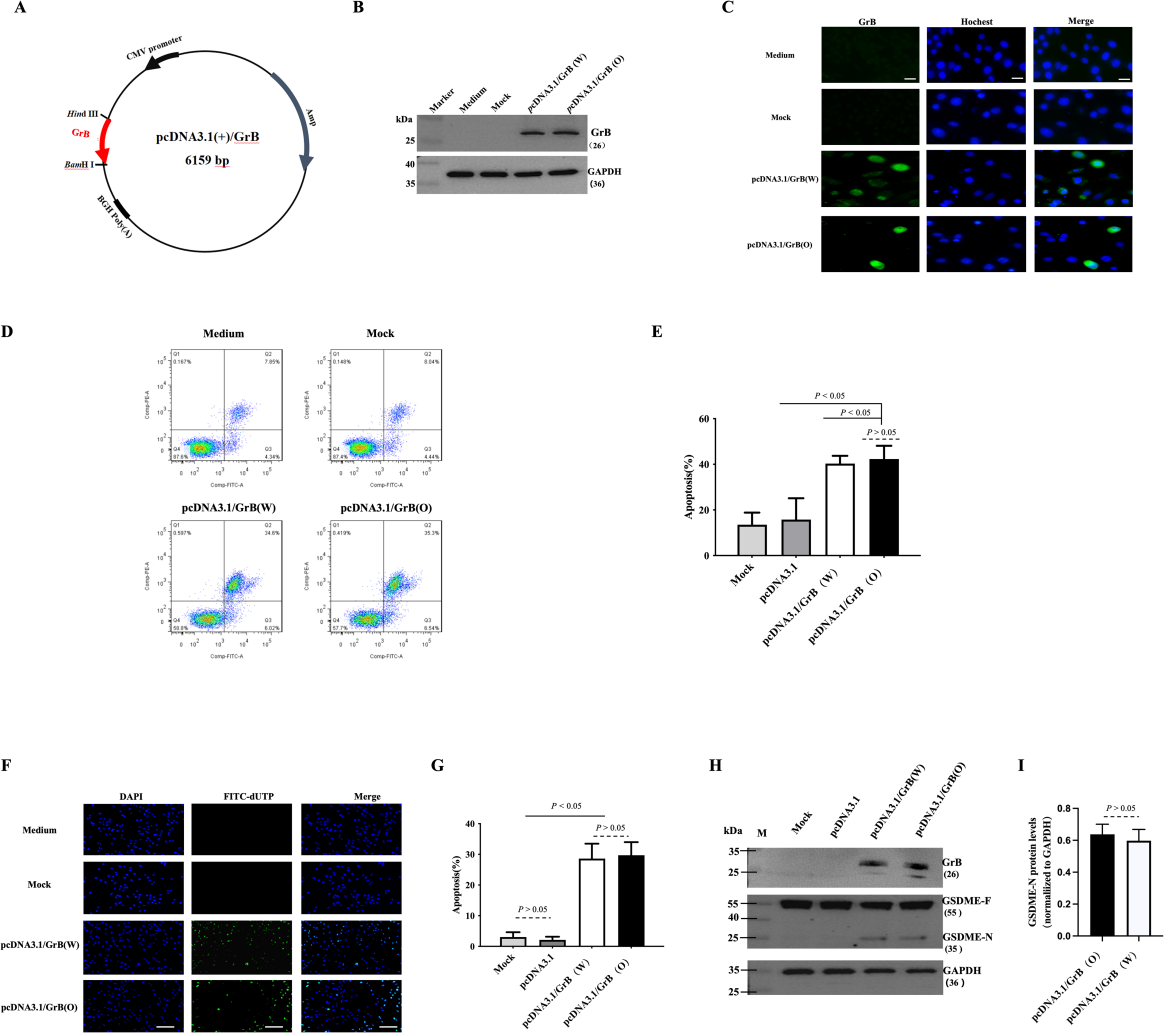
Biological activity of optimized GrB. **(A)** Construction of the *p*cDNA3.1-GrB plasmid. **(B, C)** Expression of GrB in SiHa cells 24 hours after transfection with the *p*cDNA3.1-GrB plasmid: **(B)** Western blot analysis and **(C)** immunofluorescence staining. Mock indicates the empty plasmid control, GrB(W) represents wild-type Granzyme B, and GrB(O) represents optimized Granzyme **(B)** Scale bar = 100 μm. **(D, E)** Apoptosis analysis in transfected SiHa cells, performed by flow cytometry. **(F, G)** Apoptotic cell detection via the TUNEL assay. Scale bar = 100 μm. **(H, I)** Detection of GSDME cleavage in transfected cells by Western blot: GSDME-F (full-length) and GSDME-N (N-terminal fragment). All experiments were performed in SiHa cells 24 hours post-transfection.

The optimized GrB (GrB(O)) sequence was designed through amino acid mutation (R201K) and substitution of nonpolar amino acids (R96A, R100A, R102A, K221A, K222A, K225A, R226A), as detailed in **Table 1**. The corresponding nucleic acid sequence was synthesized and cloned into the *p*cDNA3.1 plasmid to create the *p*cDNA3.1/GrB(O) construct, as depicted in **Figure 1A**. SiHa cells were transfected with either the *p*cDNA3.1/GrB(O) plasmid or the *p*cDNA3.1/GrB(W) plasmid (wild-type GrB sequence). Post-transfection, GrB expression at the protein level was confirmed by Western blot analysis (**Figure 1B**). Immunofluorescence staining indicated the subcellular localization of GrB proteins (**Figure 1C**). Functional analyses revealed that both GrB(O) and GrB(W) induced apoptosis in transfected SiHa cells, as shown in **Figures 1D, E**. In addition to apoptosis, both constructs also promoted pyroptosis (**Figures 1F, G**), as evidenced by the cleavage of gasdermin E (GSDME), a key pyroptotic effector protein. This cleavage resulted in the generation of the active GSDME-N fragment (**Figures 1H, I**). This indicates that the optimized GrB retains its apoptotic and potential pyroptotic functionalities, as supported by the comparative analysis of both constructs.

**Table 1:** GrB amino acid sequence before and after optimization.

GrB designation	Amino acid sequence
Wild-type	IIGGHEAKPHSRPYMAYLMIWDQKSLKRCGGFLIRDDFVLTAAHCWGSSINVTLGAHNIKEQEPTQQFIPVKRPIPHPAYNPKNFSND IMLLQLE **R** KAK **R** T **R** AVQPLRLPSNKAQVKPGQTCSVAGWGQTAPLGKHSHTLQEVKMTVQEDRKCESDLRHYYDSTIELCVGDPEIK KTSFKGDSGGPLVCNKVAQGIVSYG **R** NNGMPPRACTKVSSFVHWI **KK** TM **KR** Y
Optimized type	IIGGHEAKPHSRPYMAYLMIWDQKSLKRCGGFLIRDDFVLTAAHCWGSSINVTLGAHNIKEQEPTQQFIPVKRPIPHPAYNPKNFSNDIML LQLE **A** KAK **A** T **A** AVQPLRLPSNKAQVKPGQTCSVAGWGQTAPLGKHSHTLQEVKMTVQEDRKCESDLRHYYDSTIELCVGDPEIKKTSFKG DSGGPLVCNKVAQGIVSYG **K** NNGMPPRACTKVSSFVHWI **AA** TM **AA** Y

Amino acid mutations are underlined in red; nonpolar amino acid substitutions are underlined in blue.

### Immunobinding properties of Z_HPV16E7_-GrB

**Figure 2:**
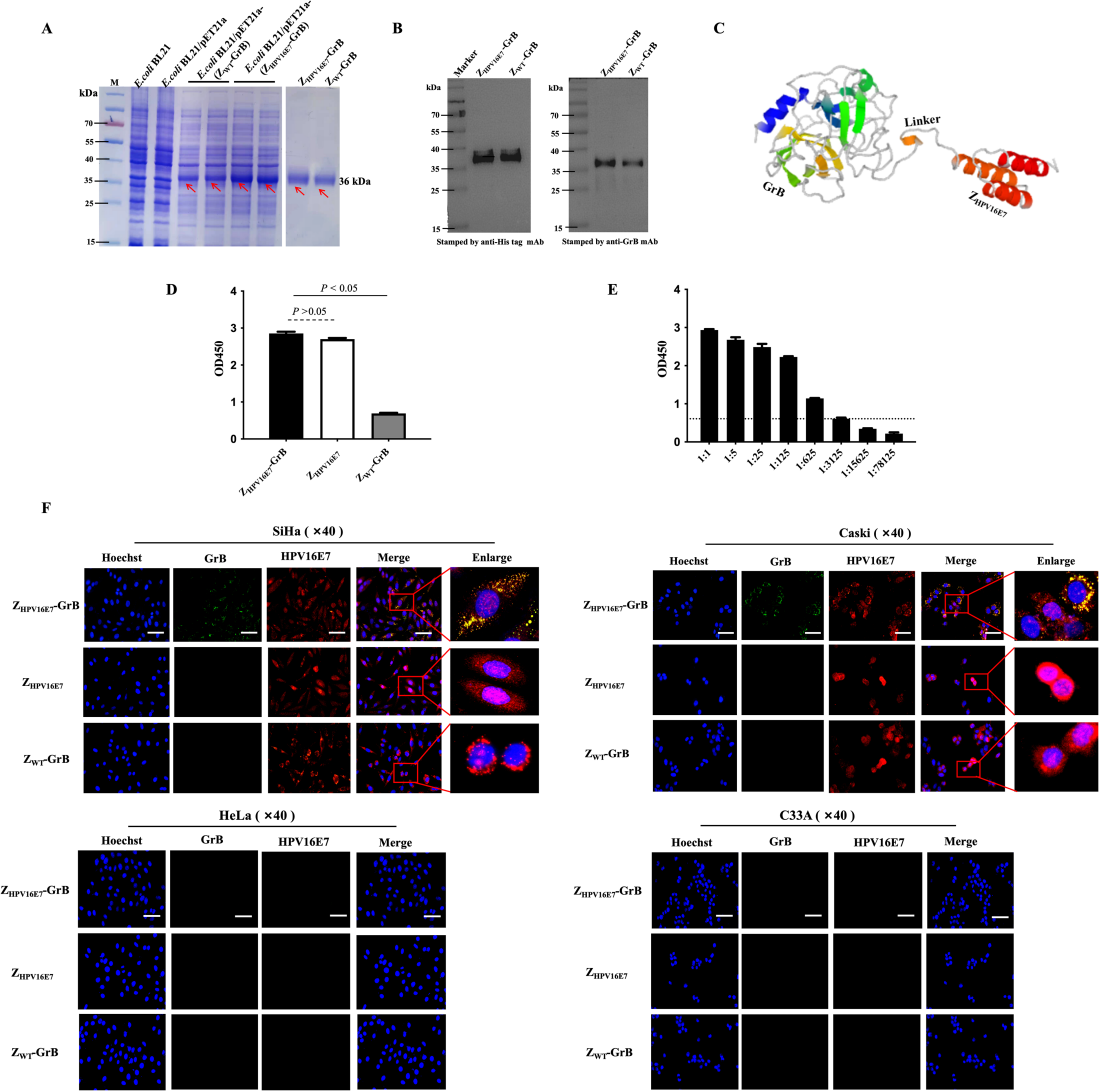
Immune affinity of Z_HPV16E7_-GrB for HPV16E7. **(A)** Prokaryotic expression and purification of Z_HPV16E7_-GrB and the nontargeting control Z_WT_-GrB (wiled type affibody-conjugated GrB without affinity screening). Red arrows indicate the purified proteins. **(B)** Western blot confirming Z_HPV16E7_-GrB binding via anti-His or anti-GrB mAbs. **(C)** Simulated 3D structure of Z_HPV16E7_-GrB, with Z_HPV16E7_ as the HPV16E7-targeting affibody and a (G_4_S)_3_ glycine-serine linker. **(D)** ELISA evaluation of Z_HPV16E7_-GrB binding to HPV16E7. **(E)** ELISA binding titer of Z_HPV16E7_-GrB to HPV16E7, with serial 1:5 dilutions and an OD_450_ cutoff of 0.5 for invalid binding. **(F)** Laser confocal microscopy showed that after 24-hour incubation with 100 μg/mL of Z_HPV16E7_-GrB, Z_HPV16E7_, or Z_WT_-GrB, Z_HPV16E7_-GrB colocalized with HPV16E7 in SiHa and CaSki (HPV16+) cells, but not in HeLa (HPV18+) or C33A (HPV-) cells. Scale bar = 100 μm.

Z_HPV16E7_-GrB was successfully expressed in the *E. coli* protein production strain, yielding a protein with a molecular weight of approximately 36 kDa (**Figure 2A**). Western blot analysis confirmed the identity of the protein through recognition by both anti-His tag and anti-GrB antibodies (**Figure 2B**). Structural modeling revealed that the Z_HPV16E7_ affibody, the flexible linker, and GrB form distinct, non-overlapping domains (**Figure 2C**), suggesting that the binding function of Z_HPV16E7_-GrB is expected to be maintained. Binding assays demonstrated that Z_HPV16E7_-GrB effectively binds to the HPV16E7 protein, confirming its targeted binding capability (**Figure 2D**). The binding titer of Z_HPV16E7_-GrB to HPV16E7 was determined to be 1:3125 (**Figure 2E**). In contrast, Z_WT_-GrB (an unscreened affibody conjugated to GrB) exhibited no binding activity. Immunofluorescence microscopy further validated the specificity of Z_HPV16E7_-GrB. Colocalization with HPV16E7 was observed in HPV16-positive SiHa and CaSki cervical cancer cells (**Figure 2F**). No colocalization was detected in HPV18-positive HeLa cells (used as a type control) or HPV-negative C33A cells (used as a negative control). The study employed four representative cell lines: HPV16-positive SiHa and CaSki for target validation, HPV18-positive HeLa to evaluate potential cross-reactivity with the distinct HPV18 E7 oncoprotein (despite shared high-risk characteristics), and HPV-negative C33A as a fundamental baseline. This strategic selection enables rigorous demonstration of Z_HPV16E7_-GrB’s exclusive specificity for HPV16 E7, as evidenced by: (1) differential responses between HPV16-positive versus HPV18-positive cells confirming E7-type discrimination, and (2) complete absence of activity in HPV-negative cells establishing target-dependence. These findings establish the specificity and high binding affinity of Z_HPV16E7_-GrB toward HPV16E7. Collectively, these results highlight the potential of Z_HPV16E7_-GrB as a targeted therapeutic agent, owing to its specificity, strong binding capability, and preserved functional domains.

### Growth inhibitory activity of Z_HPV16E7_-GrB

**Figure 3:**
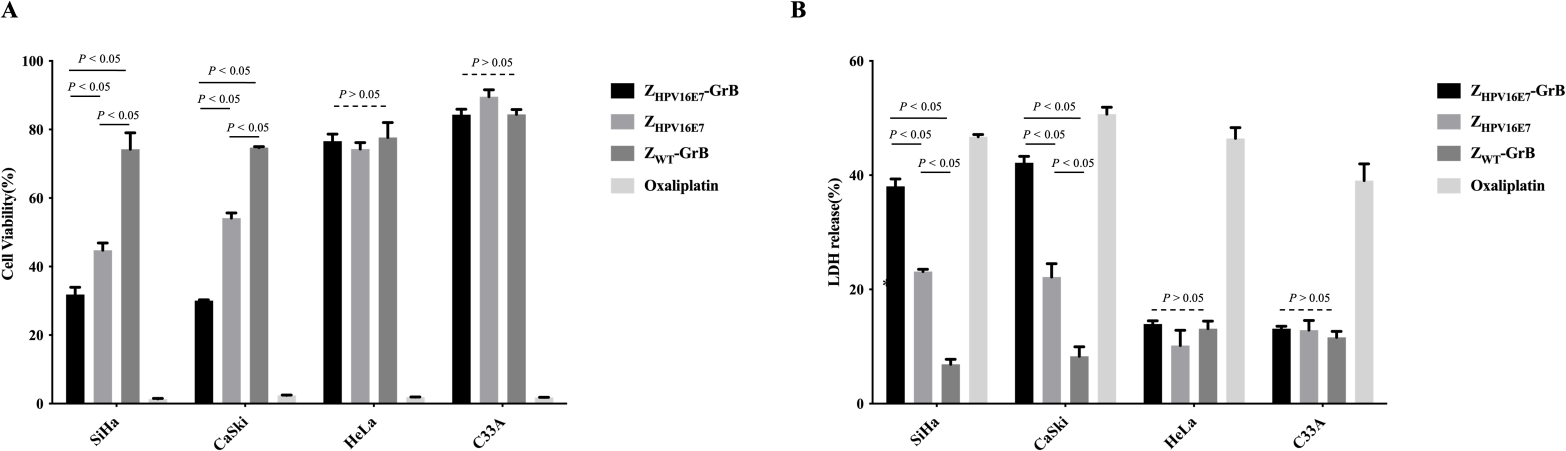
Analysis of cell growth inhibition affected by Z_HPV16E7_-GrB. **(A)** Assessment of cervical cancer cell viability using a CellTiter-Lumi™ luminescence assay after 72-hour treatment with 100 μg/mL Z_HPV16E7_-GrB, Z_HPV16E7_, Z_WT_-GrB, or 50 μg/mL oxaliplatin. **(B)** Evaluation of cell damage induced by Z_HPV16E7_-GrB through an LDH release assay following the same 72-hour treatment. Oxaliplatin was used as a positive control for cytotoxicity.

The growth-inhibitory effects of Z_HPV16E7_-GrB on HPV16-positive cells were evaluated through cell viability assays (**Figure 3A**) and lactate dehydrogenase (LDH) release, an indicator of cell damage (**Figure 3B**). Oxaliplatin, a clinically approved drug for advanced cervical cancer, was used as a positive control and demonstrated a significant reduction in cell viability alongside a marked increase in LDH release. Similarly, Z_HPV16E7_-GrB substantially reduced cell viability and significantly increased LDH release compared to Z_HPV16E7_ and Z_WT_-GrB in both SiHa and CaSki cells. In contrast, no significant reduction in cell viability or increase in LDH release was observed with Z_HPV16E7_-GrB in HeLa or C33A cells when compared to Z_HPV16E7_ and Z_WT_-GrB. These findings highlight the robust growth-inhibitory potential of Z_HPV16E7_-GrB, underscoring its promise as a targeted therapeutic agent for HPV16-associated cervical cancer.

### Z_HPV16E7_-GrB inhibits cell migration through EMT

**Figure 4:**
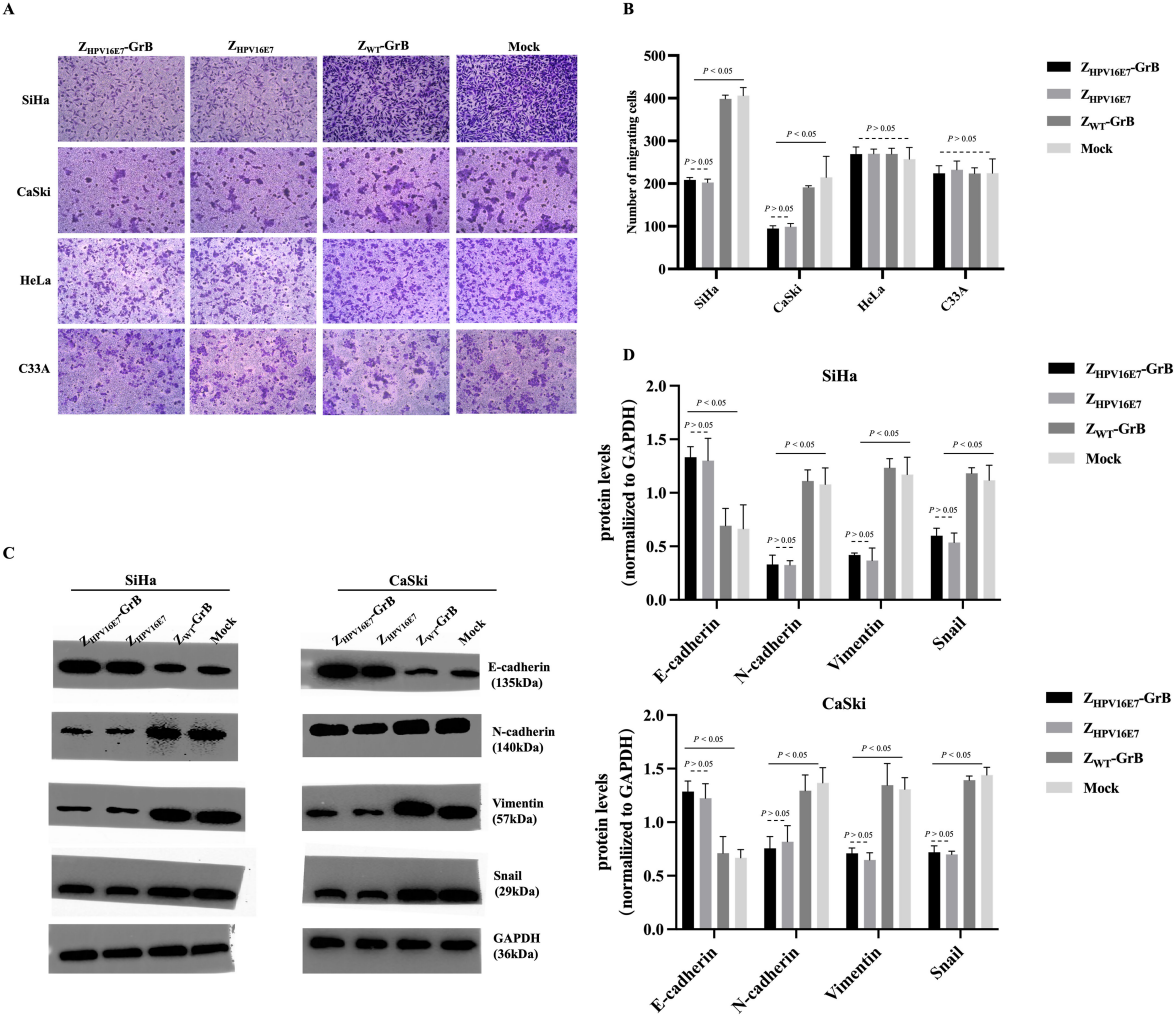
Analysis of cell migration inhibition affected by Z_HPV16E7_-GrB. **(A, B)** Transwell assay demonstrating the effect on cell migration. **(C, D)** Western blot analysis of EMT-related factors to evaluate the inhibition of epithelial-mesenchymal transition (EMT). These results were obtained after 24-hour treatment with 100 μg/mL of Z_HPV16E7_-GrB, Z_HPV16E7_, or Z_WT_-GrB.

The E7 proteins of high-risk HPV types drive the malignant transformation of infected cells through various carcinogenic mechanisms, with cancer cell migration being a key malignant behavior mediated by E7. Existing literature establishes that HPV16 E6/E7 promotes EMT through the regulation of cadherins, specifically by downregulating E-cadherin and upregulating N-cadherin expression (18). To determine whether Z_HPV16E7_-GrB could reverse this process by targeting E7, we assessed its impact on cell migration. The results showed that treatment with Z_HPV16E7_-GrB or Z_HPV16E7_ significantly inhibited cell migration, as evidenced by a reduced ability of SiHa and CaSki cells to cross the Matrigel layer, compared to equivalently treated HeLa and C33A cells (**Figures 4A, B**). Conversely, Z_WT_-GrB, which lacks specific targeting, failed to inhibit migration in all tested cell lines.

Western blot analysis of EMT-related factors (**Figures 4C, D**) revealed that treatment of SiHa and CaSki cells with Z_HPV16E7_-GrB or Z_HPV16E7_ significantly altered the expression of key proteins governing cell migration and metastasis; specifically, both treatments caused a notable decrease in the mesenchymal regulators Vimentin and Snail (essential for EMT and associated with enhanced migration/metastasis) while significantly upregulating E-cadherin (a key protein maintaining intercellular adhesion and restricting migration); concurrently, expression of N-cadherin, another key cadherin involved in cell adhesion, was significantly decreased, resulting in a coordinated shift characterized by reduced N-cadherin and elevated E-cadherin – a reversal of the “cadherin switch” hallmark of EMT (26), where N-cadherin and E-cadherin play opposing yet interconnected roles in adhesion, tissue architecture, and metastasis; this dynamic shift, combined with the reduction in Vimentin and Snail, strongly indicates that Z_HPV16E7_-GrB and Z_HPV16E7_ inhibit the EMT process; in contrast, Z_WT_-GrB-treated cells exhibited minimal changes in these factors, confirming that the observed EMT inhibition and cadherin expression reversal are direct consequences of HPV16 E7 targeting; collectively, these findings demonstrate that Z_HPV16E7_ and Z_HPV16E7_-GrB effectively inhibit the migration and invasion of HPV16-positive cervical cancer cells by E7-targeted disruption of EMT, likely through E7-targeted disruption of the EMT process.

### Induction of apoptosis by Z_HPV16E7_-GrB

**Figure 5:**
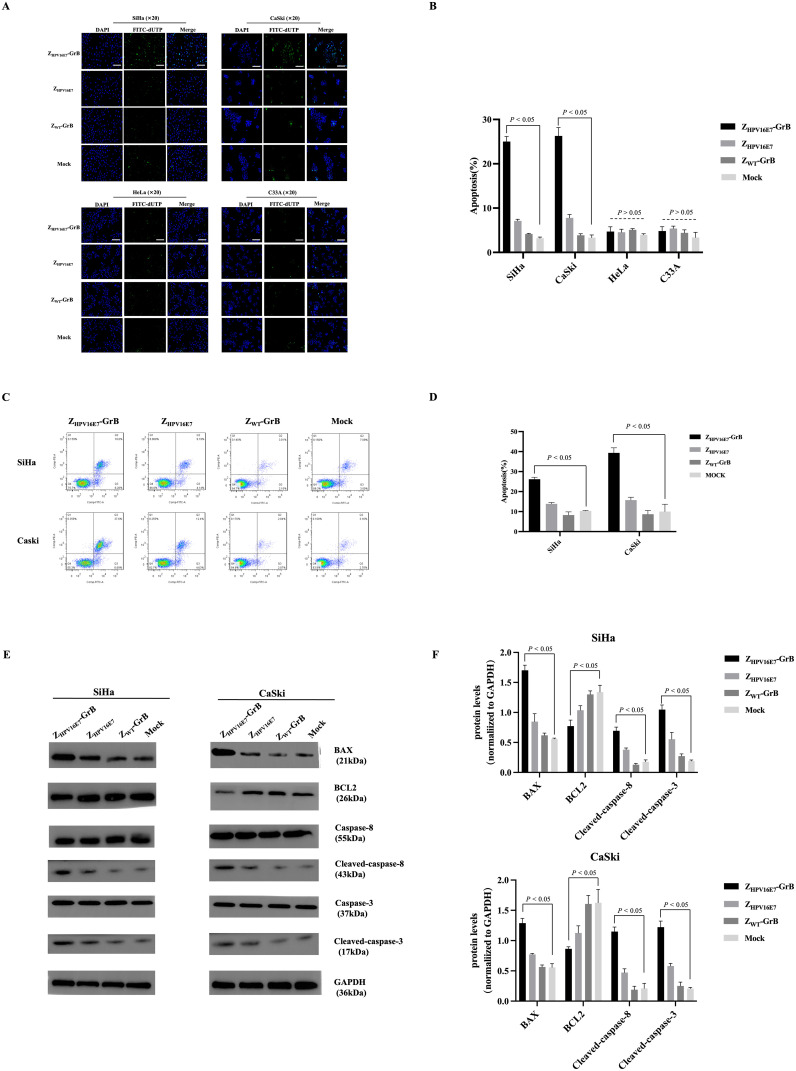
Analysis of cell apoptosis affected by Z_HPV16E7_-GrB. **(A, B)** TUNEL assay for apoptotic cell detection and quantitative analysis performed in SiHa, CaSki, HeLa, and C33A cells. Scale bar = 100 μm. **(C, D)** Flow cytometry with Annexin V/PI staining was used to assess apoptosis. **(E, F)** Western blot analysis of apoptosis-related proteins in SiHa and CaSki cells. All results were obtained from cells treated with 100 μg/mL Z_HPV16E7_-GrB, Z_HPV16E7_, or Z_WT_-GrB for 18 hours.

GrB is an immune effector molecule that induces cell death via the apoptotic pathway. In this study, the ability of Z_HPV16E7_-GrB to induce apoptosis in cervical cancer cells was assessed using TUNEL assays (**Figures 5A, B**) and flow cytometry (**Figures 5C, D**, Annexin V/PI staining). The results demonstrated that Z_HPV16E7_-GrB significantly induced apoptosis in HPV16-positive SiHa and CaSki cells, whereas no significant apoptosis was observed in the control HeLa and C33A cells.

Western blot analysis (**Figures 5E, F**) further validated these findings. In Z_HPV16E7_-GrB-treated SiHa and CaSki cells, apoptosis was induced through the activation of caspase-3 and caspase-8. This was accompanied by a significant upregulation of the pro-apoptotic protein Bax, while the anti-apoptotic protein Bcl-2 was markedly downregulated. These results indicate that Z_HPV16E7_-GrB effectively promotes apoptosis in HPV16-positive cervical cancer cells, highlighting its potential as a targeted therapeutic agent for HPV16-associated malignancies.

### Induction of pyroptosis by Z_HPV16E7_-GrB

**Figure 6:**
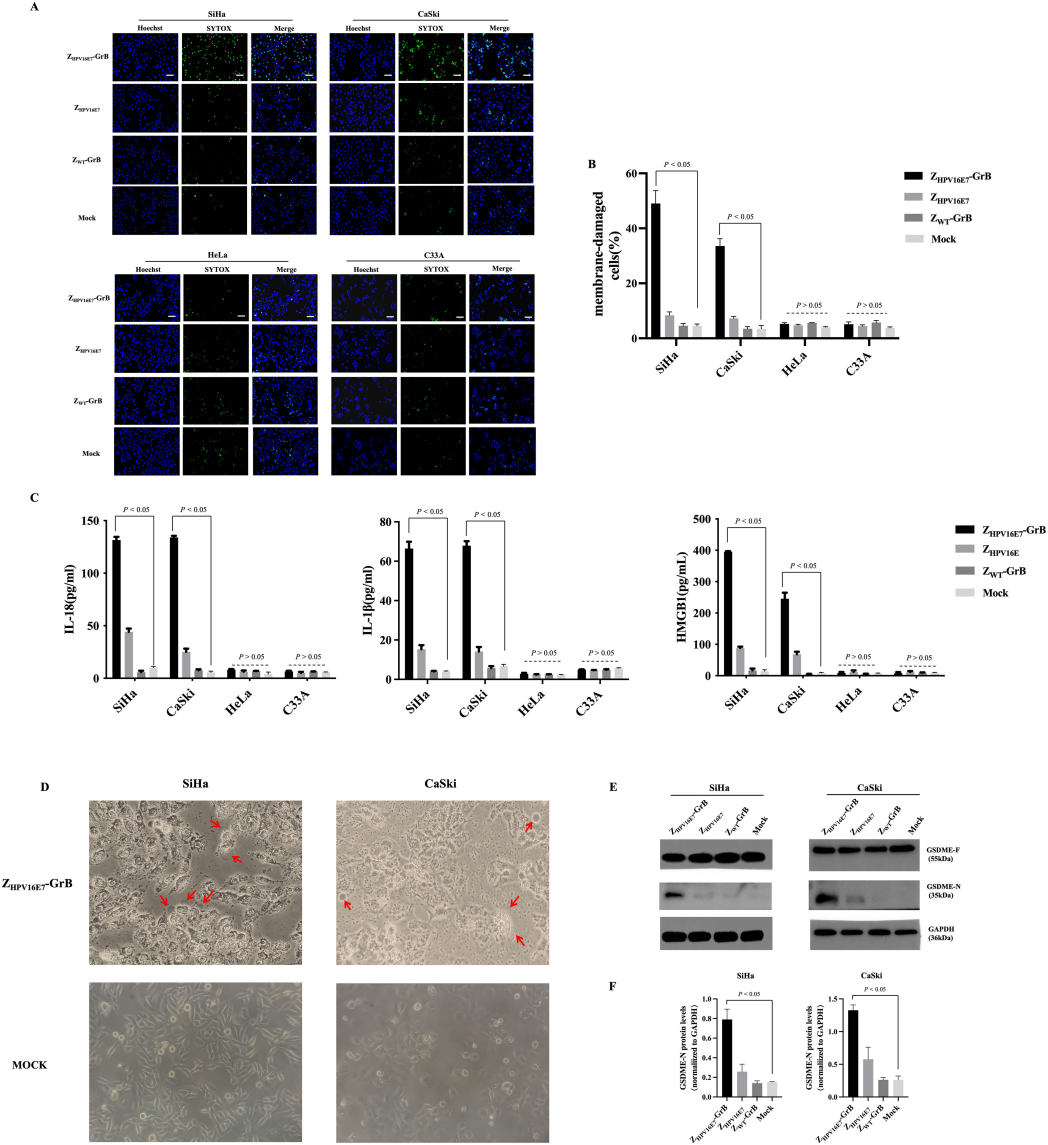
Analysis of cell pyroptosis induced by Z_HPV16E7_-GrB. **(A, B)** SYTOX Green uptake in SiHa, CaSki, HeLa, and C33A cells, indicating membrane permeability changes. Scale bar = 100 μm. **(C)** Quantification of inflammatory factor release using ELISA kits. **(D)** Light microscopy observation of membrane blebbing in SiHa and CaSki cells, with red arrows highlighting swollen cells. **(E, F)** Western blot detection of the active GSDME-N fragment in SiHa and CaSki cells. All results were obtained from cells treated with 100 μg/mL Z_HPV16E7_-GrB, Z_HPV16E7_, or Z_WT_-GrB for 24 hours.

Recent studies have shown that in addition to inducing apoptosis, GrB can also trigger pyroptosis. To investigate whether Z_HPV16E7_-GrB induces pyroptosis in HPV16-positive cervical cancer cells, we used SYTOX Green, a membrane-impermeable dye, to evaluate its effects. Z_HPV16E7_-GrB treatment resulted in a significant increase in SYTOX Green fluorescence in SiHa and CaSki cells (**Figures 6A, B**), while no noticeable changes were observed in HeLa or C33A cells. Additionally, Z_HPV16E7_-GrB treatment led to a marked release of inflammatory cytokines, including IL-18, IL-1β, and HMGB1, in SiHa and CaSki cells (**Figure 6C**), this release corresponded to the increase in SYTOX Green fluorescence, further confirming plasma membrane damage. In contrast, no significant release of inflammatory cytokines was detected in HeLa or C33A cells, confirming the HPV16-specificity of pyroptosis induction. We also assessed the key cytological features of pyroptosis in HPV16-positive cervical cancer cells. Under a light microscope, Z_HPV16E7_-GrB-treated SiHa and CaSki cells exhibited distinct membrane bubbling (**Figure 6D**, red arrows), a hallmark of pyroptosis.

Western blot analysis (**Figure 6E, F**) further validated these findings. Gasdermins, including GSDME, are critical mediators of pyroptosis. Upon cleavage, their N-terminal fragments form membrane pores, resulting in cell lysis and pyroptotic cell death. GSDME consists of an N-terminal (GSDME-N) and a C-terminal (GSDME-C) domain and primarily exists in its inactive full-length form (GSDME-F). Proteolytic cleavage of GSDME-F releases the active GSDME-N fragment, which integrates into the cell membrane, compromising its integrity. This process plays a pivotal role in tumor suppression and immune responses. Consistently, the cleaved GSDME-N fragment (35 kDa) was detected in SiHa and CaSki cells treated with Z_HPV16E7_-GrB. These findings demonstrate that Z_HPV16E7_-GrB effectively induces pyroptosis in HPV16-positive cervical cancer cells, unveiling a novel cell death mechanism with potential therapeutic applications.

### The pyroptosis pathway induced by Z_HPV16E7_-GrB

**Figure 7:**
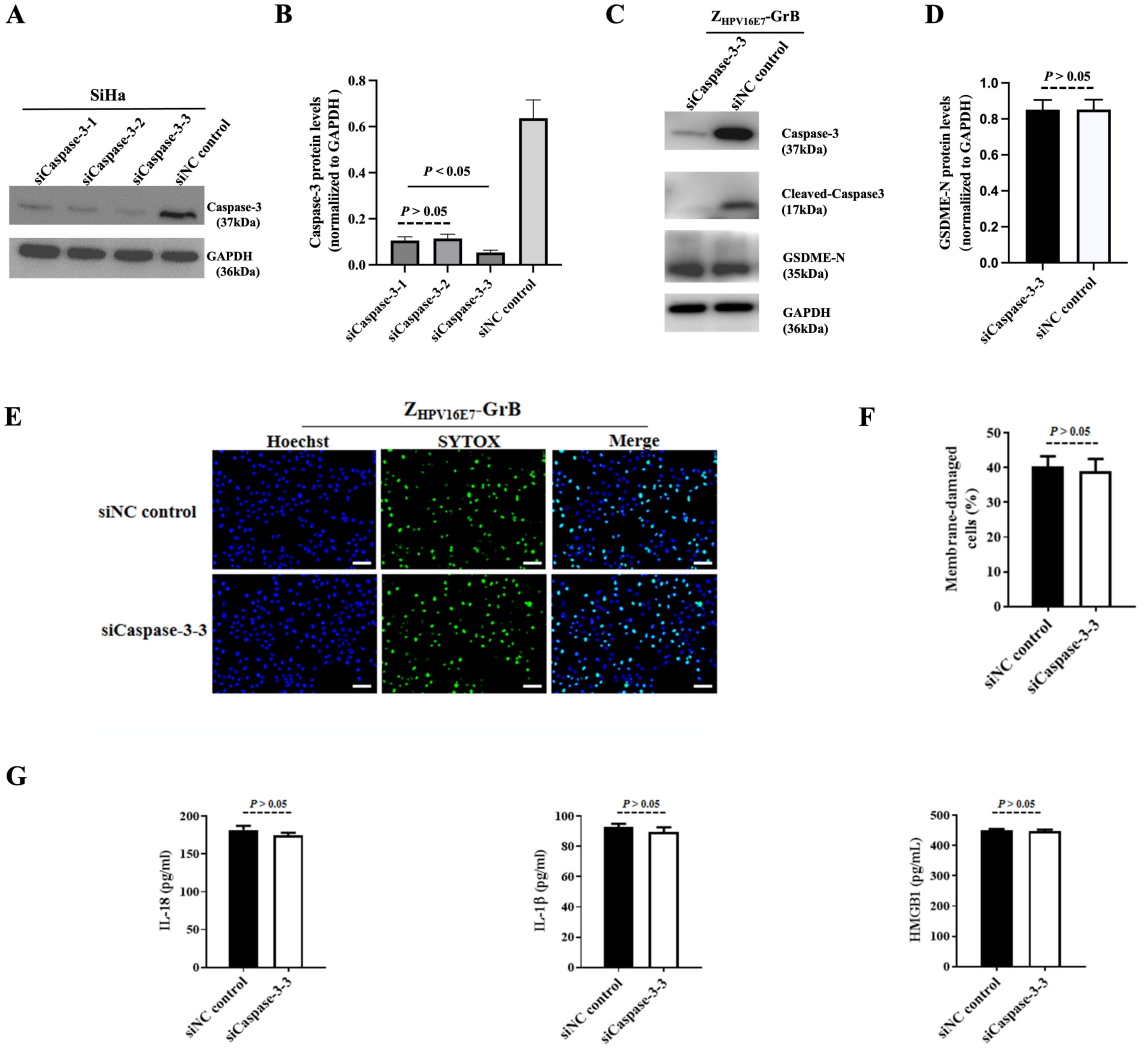
Pyroptosis analysis following caspase-3 knockdown. **(A)** Verification of caspase-3 knockdown in SiHa cells using siRNA. **(B, C)** Detection of cleaved GSDME in SiHa cells after caspase-3 knockdown. **(D, E)** Pyroptosis of SiHa cells after caspase-3 knockdown, treated with 100 μg/mL Z_HPV16E7_-GrB, observed by uptake of the membrane-impermeable dye SYTOX Green. Scale bar = 100 μm. **(F)** Quantification of inflammatory factor release in SiHa cells under the same treatment conditions as in **(D, E)**.

Granzyme-induced pyroptosis is traditionally understood to occur through the caspase-3-dependent cleavage of GSDME. However, recent research suggests that GrB can also directly induce pyroptosis by cleaving GSDME in a caspase-3-independent manner. To investigate whether Z_HPV16E7_-GrB cleaves GSDME via this alternative pathway, we conducted a caspase-3 interference experiment. Specific siRNAs targeting caspase-3 were transfected into SiHa cells, significantly suppressing caspase-3 expression, with siCaspase-3–3 showing the strongest inhibitory effect (**Figures 7A, B**), achieving over 93% knockdown efficiency.

Even with caspase-3 knockdown, cleaved GSDME-N fragments were still detected in Z_HPV16E7_-GrB-treated SiHa cells, with band intensity comparable to that of the siNC control group (**Figures 7C, D**). Additionally, Z_HPV16E7_-GrB continued to induce apoptosis, as evidenced by the SYTOX green fluorescence signals, which were similar to those observed in the siNC control group (**Figures 7E, F**). Moreover, the levels of released inflammatory cytokines IL-18, IL-1β, and HMGB1 were also comparable to those in the siNC control group (**Figure 7G**). These findings suggest that Z_HPV16E7_-GrB can induce pyroptosis through the direct cleavage of GSDME, independent of caspase-3 activity.

### Z_HPV16E7_-GrB directly cleaves GSDME

**Figure 8:**
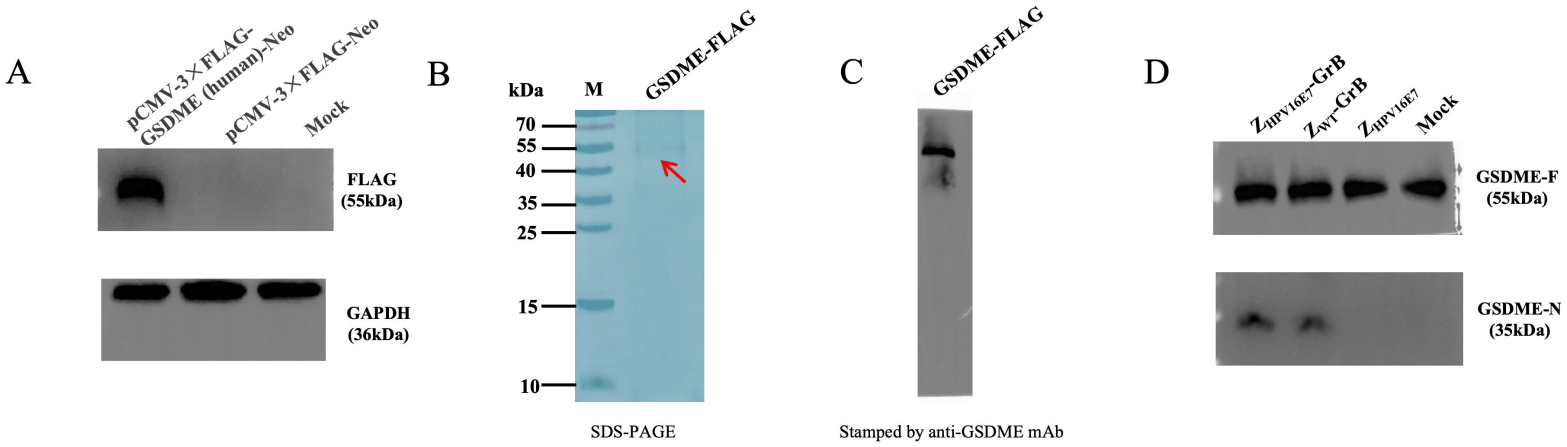
Z_HPV16E7_-GrB directly cleaves GSDME. **(A)** Expression of GSDME-FLAG in SiHa cells, detected by anti-FLAG antibody. **(B)** Purification of recombinant GSDME-FLAG (from A) via affinity chromatography. **(C)** Western blot analysis of purified GSDME-FLAG using anti-GSDME antibody. **(D)** For the *in vitro* cleavage assay, purified FLAG-GSDME (100 μg) was incubated with equimolar amounts (100 μg) of Z_HPV16E7_-GrB, Z_WT_-GrB, or Z_HPV16E7_ in reaction buffer at 37°C for 1 hour. GSDME cleavage was then analyzed by Western blot using anti-GSDME antibody.

Recent studies have demonstrated that GrB can induce pyroptosis through direct cleavage of GSDME (22). To investigate whether Z_HPV16E7_-GrB utilizes this mechanism, we engineered a eukaryotic expression system for FLAG-tagged human GSDME. Following transient transfection in SiHa cells, FLAG-GSDME expression was confirmed by anti-FLAG Western blot (**Figure 8A**), with subsequent purification via anti-FLAG affinity chromatography yielding a predominant 55-kDa band corresponding to full-length GSDME (**Figure 8B**), which was specifically recognized by anti-GSDME antibodies (**Figure 8C**).

*In vitro* cleavage assays revealed that both Z_HPV16E7_-GrB and Z_WT_-GrB—but not the affibody control Z_HPV16E7_—efficiently cleaved GSDME (**Figure 8D**), demonstrating that Z_HPV16E7_-GrB induces pyroptosis in HPV16-positive cells through direct cleavage of GSDME by GrB.

## Discussion

Current treatment strategies for cervical cancer often struggle to effectively eliminate HPV-infected cancer cells. While prophylactic HPV vaccines have significantly reduced infection rates, they provide no benefit to individuals with established infections or HPV-associated malignancies (27). Therapeutic vaccines, designed to stimulate immune responses against HPV oncoproteins such as E6 and E7, have shown promise in preclinical and early clinical studies. However, their efficacy in advanced tumors is frequently compromised by the immunosuppressive tumor microenvironment and insufficient immune activation (28). Similarly, immune checkpoint inhibitors, such as PD-1/PD-L1 and CTLA-4 inhibitors, can enhance anti-tumor immunity by reversing T-cell exhaustion, yet their overall response rates remain low, with only a subset of patients experiencing meaningful clinical benefits, particularly in recurrent or metastatic cervical cancer (29). To overcome these challenges, we developed and evaluated Z_HPV16E7_-GrB, a bifunctional therapeutic designed to enhance specificity and efficacy while minimizing off-target effects. This construct integrates the high affinity of Z_HPV16E7_ for HPV16-positive cells with the potent cytotoxic activity of GrB, enabling a targeted approach for more effective treatment.

Immunotoxins, often referred to as “targeting missiles,” represent a promising strategy in cancer therapy due to their ability to precisely deliver toxic effectors into tumor cells, leading to cell death. These conjugates typically combine monoclonal antibodies with toxin molecules to ensure targeted delivery (30). At present, the majority of toxin molecules used in immunotoxins are derived from exogenous sources such as bacteria or plants, but the potential immunogenicity caused by the heterogeneity hampers their clinical use (31). To overcome this limitation, our laboratory has developed immunotoxins incorporating endogenous cytotoxic molecules, with a particular focus on Granzyme B (GrB). To enhance its therapeutic potential, we genetically engineered GrB to minimize non-specific binding to cells while preserving its potent cytotoxic activity. In our study, we compared the functional properties of the optimized GrB with its wild-type counterpart. Our findings revealed that both wild-type and engineered GrB effectively induce significant apoptosis in tumor cells. Notably, apoptosis is not the sole mechanism of cell death triggered by these immunotoxins. GrB has the ability to cleave GSDME, triggering pyroptosis—a pro-inflammatory form of programmed cell death. Both wild-type and optimized GrB have been shown to cleave GSDME, resulting in the generation of the active GSDME-N fragment that initiates the pyroptotic process.

As promising delivery molecules, affibodies with a molecular weight of only approximately 6.5 kDa, offer excellent permeability, weak immunogenicity, and ease of preparation, making them theoretically superior to mAbs in terms of targeted delivery (32). The core of tumor-targeted therapy lies in its ability to precisely target cancer-specific molecules, including those in metastatic cells, to induce cell death or dysfunction (33). Therapeutic strategies targeting HPV E7 have demonstrated significant potential in the treatment of cervical cancer (34). Thus, the combination of targeted inhibiting oncogenic pathways and inducing cell death is the key strategy to tumor targeted therapy. From a technological platform perspective, the affibody-based Z_HPV16E7_-GrB and monoclonal antibody-based antibody-drug conjugates (ADCs) such as Trastuzumab-GrB exhibit fundamental differences that are primarily manifested in their molecular size and tissue penetration capabilities, with the smaller affibody format demonstrating superior tumor penetration and intracellular delivery efficiency compared to conventional ADCs, while importantly both systems maintain comparable efficacy in inducing apoptosis in target cells (35).

Our findings demonstrated that Z_HPV16E7_-GrB, expressed in a prokaryotic system, retained strong immune recognition and exhibited high binding specificity for HPV16E7 and HPV16-positive cancer cells, such as SiHa and CaSki. Notably, it showed no binding to non-HPV16-positive cells, including HeLa and C33A, and no unintended effects were observed. This precise targeting design effectively minimizes off-target effects while maximizing tumor specificity. Additionally, Z_HPV16E7_-GrB significantly reduced cell viability and increased LDH release in these cell lines, confirming its cytotoxic activity. We propose that the inhibitory effect on cell growth stems from a dual mechanism: (i) Z_HPV16E7_ binds to the oncogenic E7 protein, blocking its cancer-promoting function, and (ii) GrB exerts strong cytotoxic effects through targeted delivery by Z_HPV16E7_.

The clinical management of HPV-associated cervical cancer remains challenging due to high recurrence and metastasis rates, poor prognosis, and resistance to conventional therapies. Among these challenges, recurrence and metastasis are particularly concerning, as they are the primary drivers of cervical cancer-related mortality (36, 37). During persistent high-risk HPV infection, the viral oncoprotein E7 promotes EMT through multiple mechanisms, including the disruption of E-cadherin expression, upregulation of N-cadherin, and activation of EMT-inducing transcription factors. These processes contribute significantly to tumor recurrence and metastasis (38–40). Therefore, targeting the E7-EMT pathway represents a promising therapeutic strategy to suppress tumor invasiveness and metastasis, ultimately improving the overall prognosis of patients with advanced or recurrent disease. In our study, both the immunoaffinity toxin Z_HPV16E7_-GrB and Z_HPV16E7_ alone effectively inhibited the migration of HPV16-positive SiHa and CaSki cervical cancer cells. This inhibition was evidenced by a significant reduction in the expression of EMT markers Vimentin and Snail, a marked increase in E-cadherin expression, and a notable decrease in N-cadherin expression. These molecular changes indicate a reversal of the EMT process, potentially limiting the invasive and metastatic capabilities of cervical cancer cells. These findings underscore the potential of Z_HPV16E7_-GrB and Z_HPV16E7_ to target HPV E7, regulate EMT, and suppress cervical cancer progression. By addressing the critical mechanisms underlying metastasis, these agents offer a promising therapeutic strategy for the treatment of advanced and recurrent HPV16-positive cervical cancer.

The perforin- granzyme mechanism is a well-established pathway employed by cytotoxic immune cells of CTLs and NK cells, to induce apoptosis in target cells (41). Interestingly, the specific targeting cytotoxicity mediated by the immunoaffitoxin Z_HPV16E7_-GrB appears to closely mimic this process. Another exciting discovery is that Z_HPV16E7_-GrB also induced pyroptosis, a pro-inflammatory form of programmed cell death, standing out for amplifying anti-tumor immunity. Unlike apoptosis, pyroptosis results in membrane rupture, releasing intracellular contents and triggering inflammation. Inflammasome-independent pyroptosis has been observed in tumor cells under conditions such as hypoxia, exposure to chemotherapeutic drugs, or gasdermin cleaved by immune effectors (42–45). For instance, a research report showed that granzyme A (GrA) could cleave gasdermin B (GSDMB, one of members in gasdermin family) to induce pyroptosis, thereby enhancing anti-tumor immunity (46). Another research showed that chemotherapeutic agents in gastric cancer and small-molecule kinase inhibitors in lung cancer and melanoma cells also induced pyroptosis through the GSDME/caspase-3 pathway to contribute significantly on tumor suppression (47). Our study provides compelling evidence that the immunoaffitoxin Z_HPV16E7_-GrB induces not only apoptosis, a classical mechanism of programmed cell death mediated through caspase-3 activation, but also pyroptosis, characterized by the production of GSDME-N through direct cleavage of GSDME, cell swelling, membrane rupture, and the release of pyroptosis-related factors, further enhancing its therapeutic potential. The joint cell death of apoptosis and pyroptosis highlights the potential of Z_HPV16E7_-GrB as a powerful tool in cancer immunotherapy, and especially pyroptosis, with its ability to provoke robust immune activation, may serve as a complementary mechanism to apoptosis, further amplifying the anti-tumor immune response. These findings provide a strong explanation for our previous research, which demonstrated the potent anti-tumor effects of Z_HPV16E7_-GrB in an HPV16-positive cervical cancer mouse model (25).

The key advantage of GrB over plant/bacterial toxins is its human origin, which minimizes immunogenicity (48). This contrasts with traditional immunotoxins, where neutralizing antibodies against non-human toxin domains often hinder clinical translation. While the affibody Z_HPV16E7_ is also predicted to exhibit low immunogenicity (49), empirical validation of the Z_HPV16E7_-GrB fusion protein remains essential. Beyond immunogenicity, *in vitro* studies demonstrate that Z_HPV16E7_-GrB’s precision-targeting design eliminates off-target effects, but its systemic safety profile requires further evaluation—particularly *in vivo* off-target toxicity. Pharmacokinetic properties also require thorough investigation: While Z_HPV16E7_-GrB itself remains unevaluated, preliminary data show that unconjugated Z_HPV16E7_ (administered intravenously in mice) is eliminated within 100 hours (17)—significantly faster than monoclonal antibodies (~100 days) (50). Thus, optimizing the half-life of Z_HPV16E7_-GrB through bioengineering is essential, paralleling the need for comprehensive pharmacokinetic and safety studies.

## Conclusion

**Figure 9:**
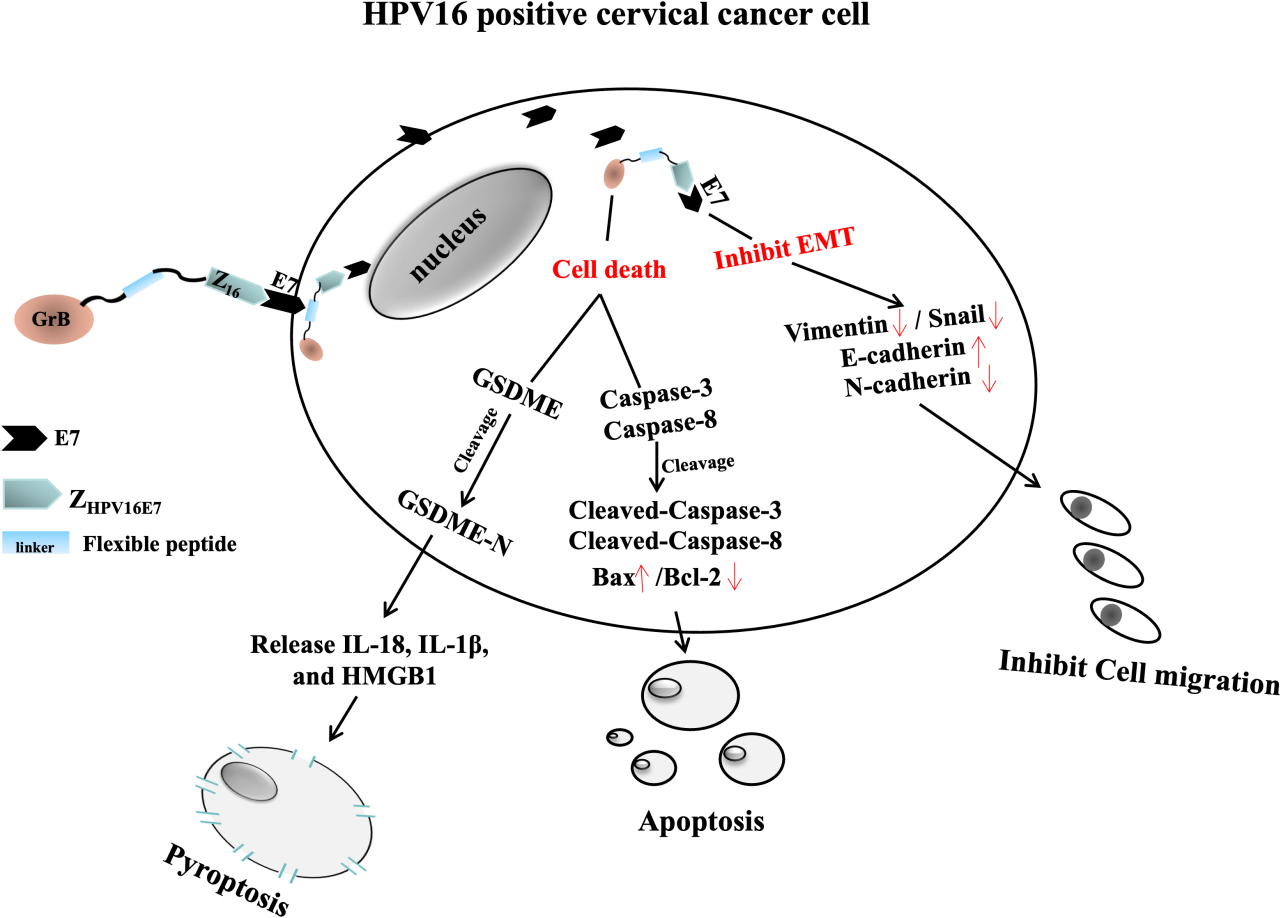
Mechanistic diagram of Z_HPV16E7_-GrB action in HPV16-positive cervical cancer cells. Z_HPV16E7_-GrB consists of Z_HPV16E7_ (targeting HPV16E7), a flexible peptide linker, and GrB. Z_HPV16E7_ inhibits the growth of HPV16-positive cervical cancer cells and suppresses EMT, as evidenced by reduced expression of Vimentin and Snail, increased expression of E-cadherin, and decreased expression of N-cadherin. GrB exerts its function by inducing two forms of programmed cell death: apoptosis and pyroptosis. It induces apoptosis via caspase-3 and caspase-8 activation, accompanied by an increase in the pro-apoptotic factor Bax and a significant reduction in the anti-apoptotic factor Bcl-2. Additionally, GrB triggers pyroptosis through direct cleavage of GSDME, independent of caspase-3 activity, leading to membrane rupture and the release of proinflammatory cytokines, including IL-18, IL-1β, and HMGB1.

In summary, our work provides a novel framework for exploring targeted anti-tumor therapies, leveraging the bi-functional anti-tumour advantage of Z_HPV16E7_-GrB, with one function mediated by Z_HPV16E7_, inhibiting the migration of HPV16+ cervical cancer cells by suppressing EMT, and the other function mediated by GrB, inducing the joint cell death including apoptosis and pyroptosis to enhance anti-tumor immunity and efficacy. This dual mechanism holds significant promise for advancing the treatment of HPV16-associated cancers. The working model of Z_HPV16E7_-GrB is shown in **Figure 9**.

## Data Availability

The original contributions presented in the study are included in the article/supplementary material. Further inquiries can be directed to the corresponding author.
